# Supplementary material for: Emergent order in the kagome Ising magnet Dy3Mg2Sb3O14
Source: Nat Commun. 2016 Dec 20;7:13842. doi: 10.1038/ncomms13842 (PMC5187434; doi:10.1038/ncomms13842)
Supplement: Supplementary Information — Supplementary Figures, Supplementary Tables, Supplementary Notes and Supplementary References. [file ncomms13842-s1.pdf]

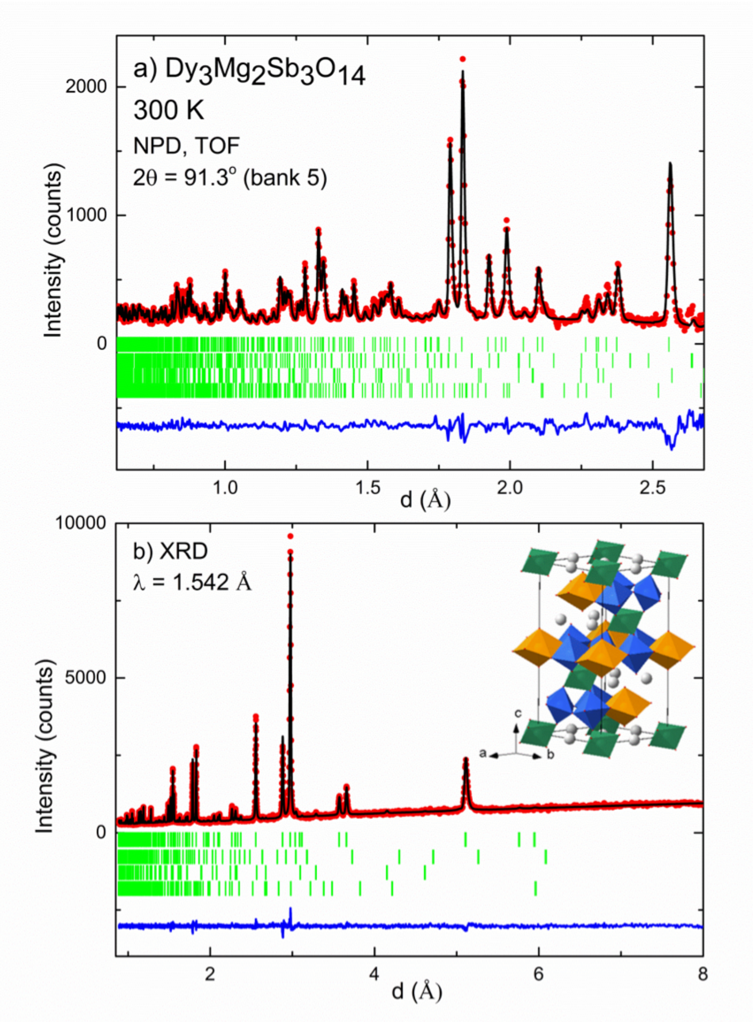

Supplementary Figure 1: **Crystal-structure refinements for $Dy_3Mg_2Sb_3O_{14}$.** Neutron (a) and X-ray (b) diffraction measurements for $Dy_3Mg_2Sb_3O_{14}$ at 300 K. Neutron data were collected using the GEM diffractometer at the ISIS neutron source. Observed intensities and calculated intensities obtained from a combined multi-bank Rietveld refinement are shown as red circles and a black line, respectively; the difference (data–fit) is shown by a blue line. Reflection positions are indicated by green tick marks, for phases (top to bottom): $Dy_3Mg_2Sb_3O_{14}$, Dy_3SbO_7, $MgSb_2O_6$, and VO_2. A polyhedral model for the structure of $Dy_3Mg_2Sb_3O_{14}$ is inset in (b). Mg1, Mg2(Dy) and Sb1 polyhedra are shown in green, orange, and blue, respectively; Dy1(Mg) sites are shown as white spheres.

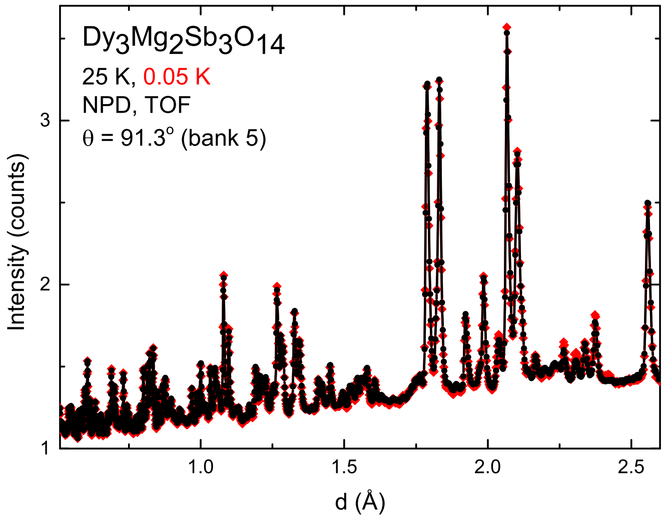

Supplementary Figure 2: **Comparison of 25 K and 0.05 K neutron-diffraction data for $Dy_3Mg_2Sb_3O_{14}$.** Neutron-diffraction data for $Dy_3Mg_2Sb_3O_{14}$ measured using the GEM diffractometer at the ISIS neutron source at 25 K (black circles) and 0.05 K (red diamonds), showing the absence of a crystallographic phase transition. We note that the magnetic Bragg peaks at 0.05 K are very weak for $d \lesssim 2$ Å, because of the magnetic form factor. The extra peaks visible here compared to Supplementary Figure 1(a) are from the low-temperature sample environment.

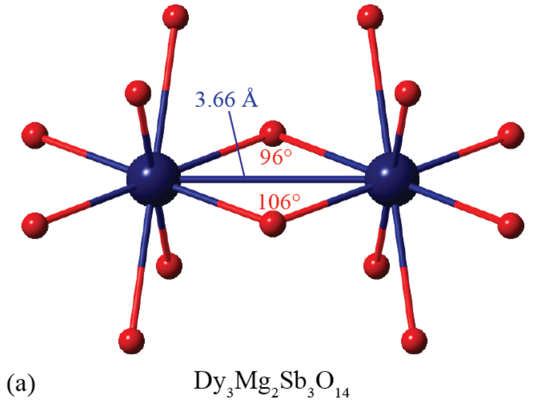

Supplementary Figure 3: **Comparison of Dy environment in $Dy_3Mg_2Sb_3O_{14}$ with $Dy_2Ti_2O_7$.** Local Dy environment in $Dy_3Mg_2Sb_3O_{14}$ (a) and $Dy_2Ti_2O_7$ (b), showing Dy (large blue spheres) and O (small red spheres). The Dy–Dy bond length and Dy–O–Dy bond angles are labelled. All values are at room temperature; values for $Dy_2Ti_2O_7$ are derived from [1].

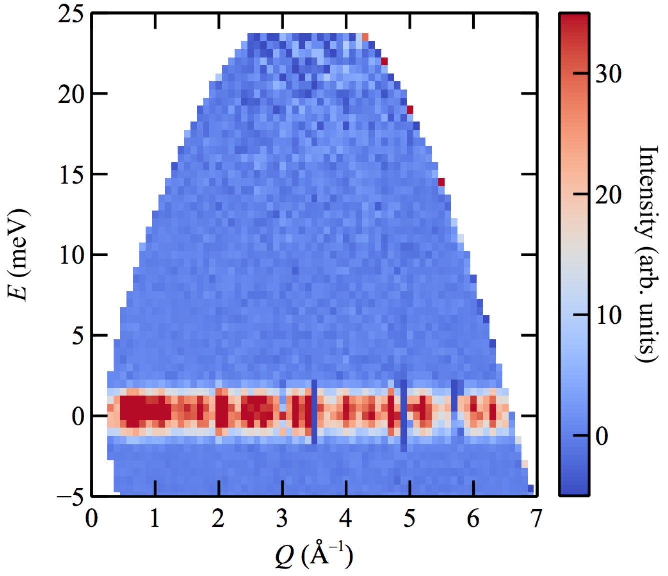

Supplementary Figure 4: **High-energy inelastic scattering for $Dy_3Mg_2Sb_3O_{14}$.** Momentum and energy dependence of neutron-scattering intensity at nominal $T = 0.03$ K, measured using the DCS instrument at the National Institute of Standards and Technology (NIST) [2] with incident neutron wavelength $\lambda = 1.8$ Å. An empty can measurement has been subtracted from the data in order to remove background scattering. These measurements reveal no crystal electric-field excitations to a maximum energy transfer 23 meV (270 K), demonstrating that the ground-state Kramers doublet is well separated from excited crystal-field states.

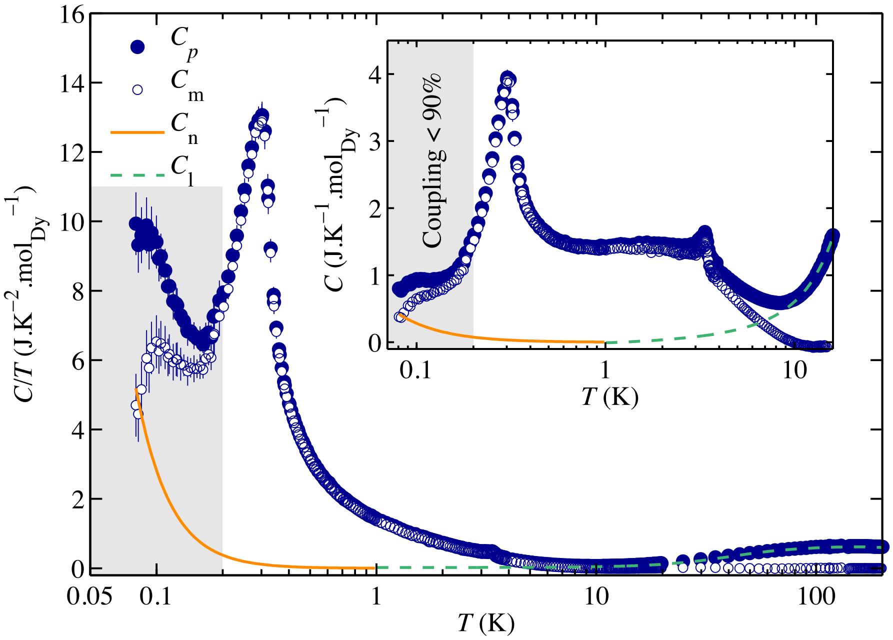

Supplementary Figure 5: **Specific-heat data processing.** Specific heat of $Dy_3Mg_2Sb_3O_{14}$, showing the following contributions: total measured (C_p, full blue circles), modelled lattice (C_l, green dashed line), modelled nuclear (C_n, solid orange line), and extracted magnetic ($C_\mathrm{m} = C_p - C_\mathrm{n} - C_\mathrm{l}$, open blue circles). The main panel shows C/T and the inset shows C. The temperature region where sample coupling falls below 90% is shaded grey. The magnetic specific heat C_m displays a very small peak at $T_i = 3.35(5)$ K, which is probably associated with the magnetic ordering of the ~ 2 wt% Dy_3SbO_7 impurity phase [3]. There is also a small peak in C_m around 0.1 K, which is of uncertain origin given the poor sample coupling below approximately 0.2 K. Error bars represent the addition of statistical and systematic uncertainties, where statistical uncertainty is calculated from a least-squares fit of the measured data to a two-timescale relaxation model, and systematic uncertainty is calculated assuming a 5% error on the sample mass.

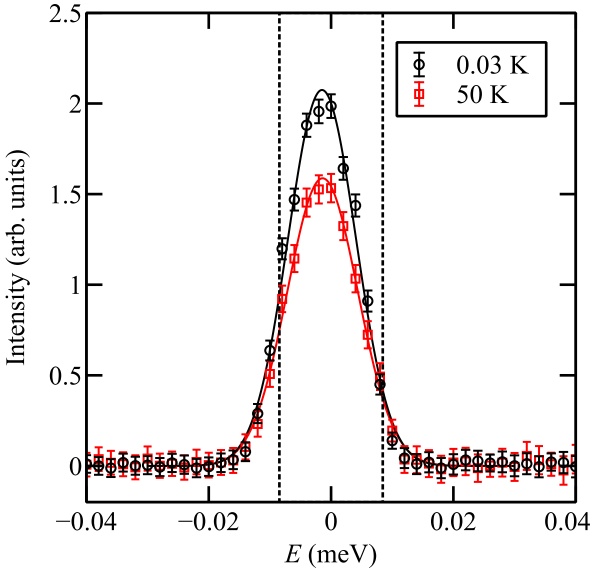

Supplementary Figure 6: **Low-energy inelastic scattering for $Dy_3Mg_2Sb_3O_{14}$.** Energy dependence of neutron-scattering intensity at 0.03 K (black circles) and 50 K (red squares), measured with incident neutron wavelength $\lambda = 10\,Å$ using the DCS spectrometer at NIST [2]. The data are integrated over the range $15 \leq 2\theta \leq 105°$, which contains no nuclear Bragg peaks but intense magnetic diffuse scattering. The black dotted vertical lines show the calculated energy resolution (FWHM) of the instrument, $16.9\,\mu$eV. The data are well described by Gaussian fits (solid lines), which yield fitted values of the FWHM of $13.1(2)\,\mu$eV at 0.03 K and $13.5(4)\,\mu$eV at 50 K; hence the energy line-width due to dynamical spin fluctuations is limited by the instrumental resolution at both temperatures. Error bars show one standard error propagated from neutron counts.

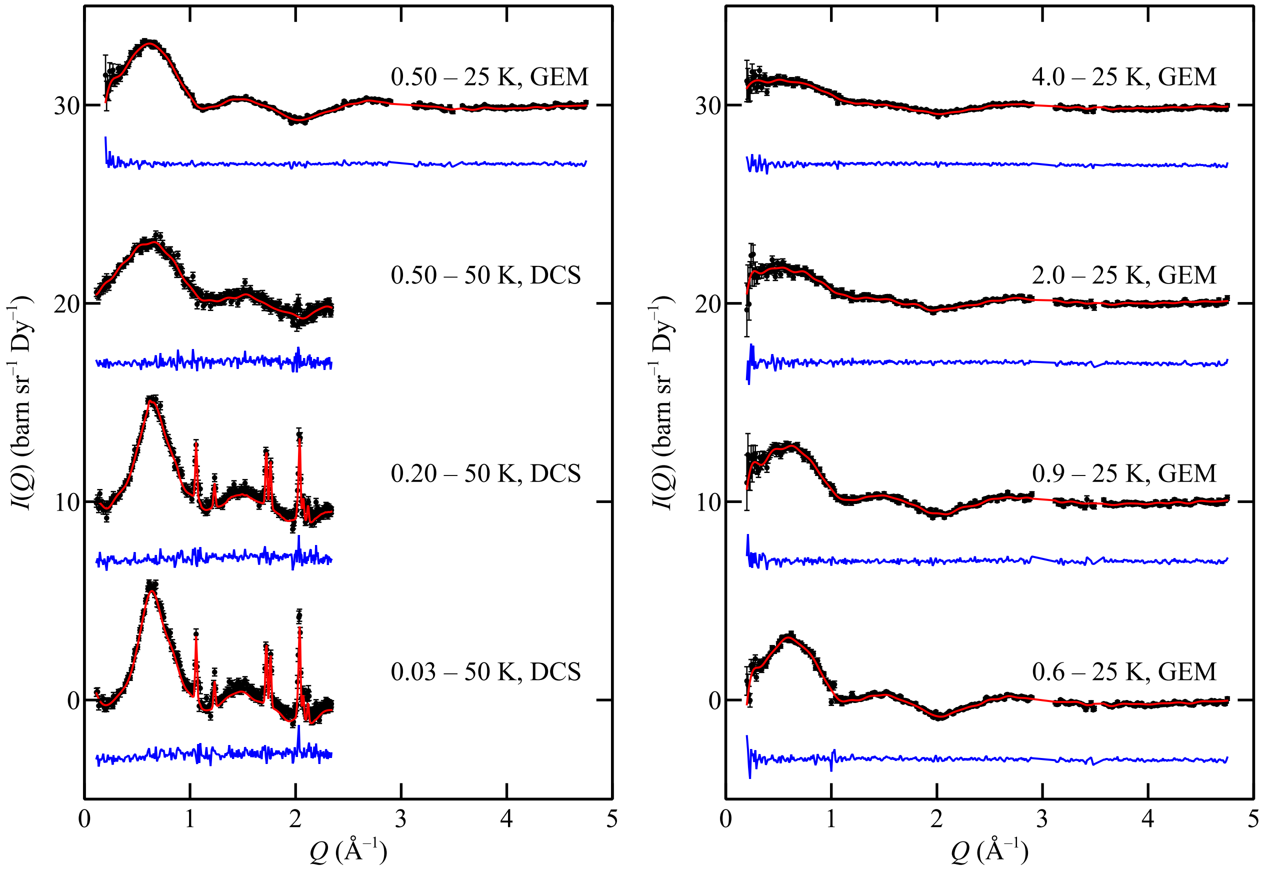

Supplementary Figure 7: **Reverse Monte Carlo fits for $Dy_3Mg_2Sb_3O_{14}$.** Representative reverse Monte Carlo fits to neutron-scattering data collected on the DCS and GEM neutron-scattering instruments, showing experimental data (black circles), RMC fits (red lines), and data–fit (blue lines). The temperature and instrument used (DCS or GEM) are labelled above each curve. Successive curves are vertically shifted by 10 barn sr^{-1} Dy^{-1} for clarity. The resulting spin configurations were used to calculate the % of triangles with ± 3 charge [Fig. 3a], and the charge correlation function [Figs. 3b and c]. Error bars show one standard error propagated from neutron counts.

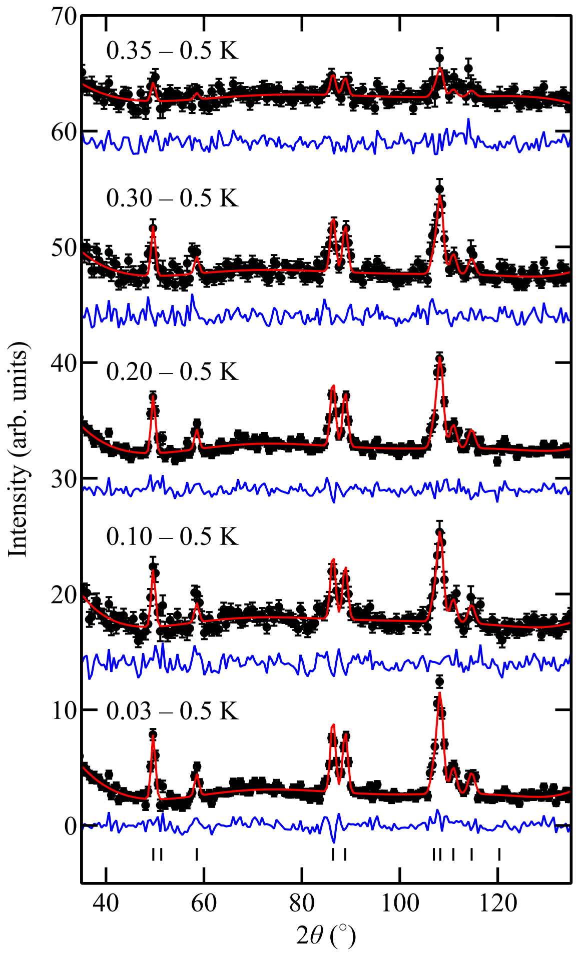

Supplementary Figure 8: **Rietveld fits for $Dy_3Mg_2Sb_3O_{14}$.** Rietveld fits to the magnetic Bragg scattering data for $Dy_3Mg_2Sb_3O_{14}$ obtained by subtracting 0.5 K data from the the 0.10, 0.20, 0.30 and 0.35 K DCS data. The incident wavelength $\lambda = 5.0$ Å. Temperatures are labelled on the graph. At each temperature, experimental data are shown as black circles, Rietveld fits as a red line, and difference (data–fit) as a blue line. Error bars show one standard error propagated from neutron counts.

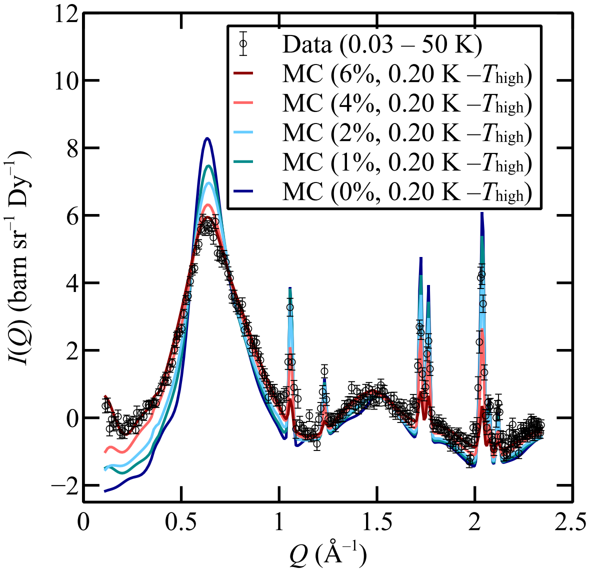

Supplementary Figure 9: **Monte Carlo neutron-scattering calculations for $Dy_3Mg_2Sb_3O_{14}$.** Powder-averaged magnetic neutron-scattering patterns calculated from Monte Carlo simulations for different amounts of random site disorder (expressed as % Mg on Dy site). Calculations (solid coloured lines) are shown for 0.2 K; experimental data collected at 0.2 K (hollow black circles) are shown for comparison, where error bars show one standard error propagated from neutron counts. Calculations are performed using the same approach as the RMC refinements (described in the Methods section), and a total magnetic moment $\mu = 10.0\,\mu_B$ per Dy is assumed. No fitting parameters are included in order to match the experimental data. Good overall agreement is achieved between 0.2 K simulations and experimental data for between $\sim$4 and 6% Mg on the Dy site; values close to 4% yield best agreement with Bragg intensities, whereas values close to 6% yield best agreement with the diffuse-scattering profile. We anticipate that the agreement may be further improved by fitting the value of the nearest-neighbour exchange interaction and/or by considering possible short-range correlations of Mg occupancy of the Dy site.

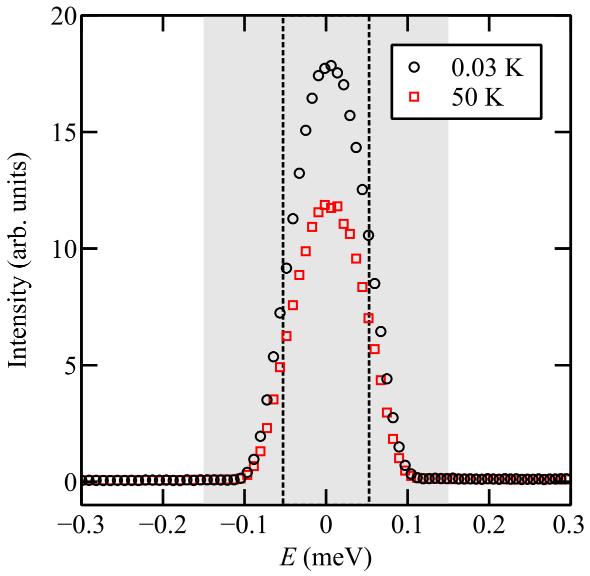

Supplementary Figure 10: **Validity of quasi-static approximation.** Energy dependence of neutron-scattering intensity at 0.03 K (black circles) and 50 K (red squares), measured with incident neutron wavelength $\lambda = 5$ Å using the DCS spectrometer at NIST [2]. The data are integrated over the range $15 \leq 2\theta \leq 45°$, which contains no nuclear Bragg peaks but intense magnetic diffuse scattering. The black dotted vertical lines show the calculated energy resolution (FWHM) of the instrument, and the grey box shows the range of energy integration for the neutron-scattering data shown in Fig. 2b. The energy line-width due to dynamical spin fluctuations is limited by the instrumental resolution at both 0.03 and 50.0 K, which demonstrates that the quasi-static approximation is ideally satisfied. The decrease in peak intensity at 50 K occurs because diffuse intensity is redistributed to higher 2θ. Standard errors (propagated from neutron counts) are smaller than the symbol size in the plots.

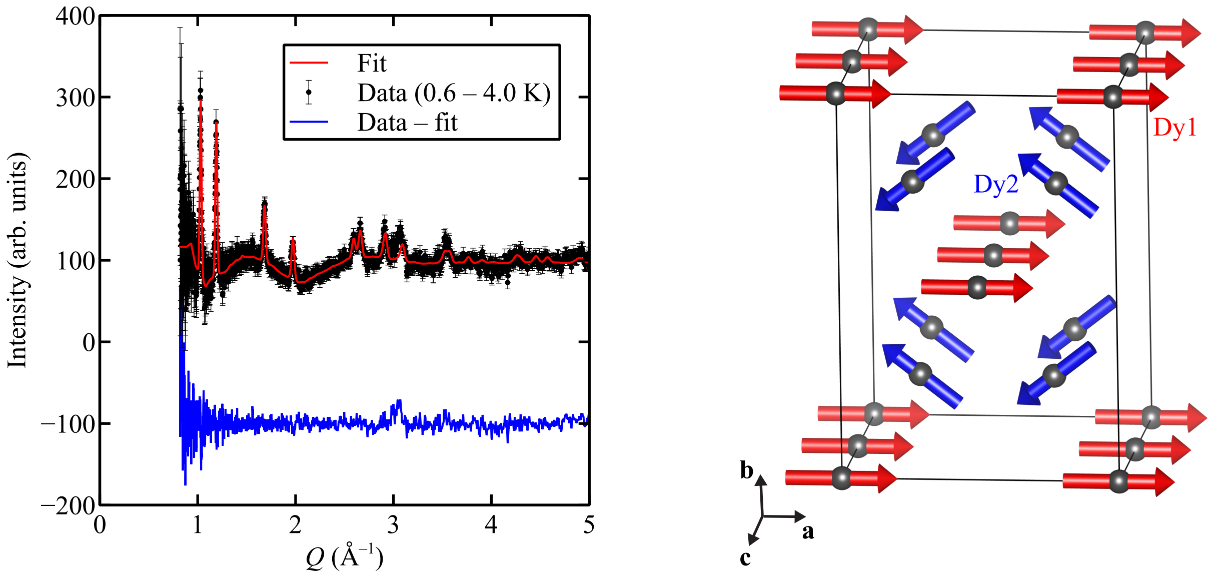

(a) (b)

Supplementary Figure 11: **Rietveld fits for Dy_3SbO_7.** (a) Rietveld fits to the magnetic Bragg scattering data for Dy_3SbO_7 obtained by subtracting 4.0 K from 0.60 K GEM data, as described in the text. Experimental data are shown as black circles, Rietveld fit as a red line, and difference (data–fit) as a blue line. (b) Model of the magnetic structure of Dy_3SbO_7 obtained from Rietveld refinement. Orientations of ordered magnetic moments are shown as red arrows (Dy1 site) and blue arrows (Dy2 site).

$Dy_3Mg_2Sb_3O_{14}$ nuclear, $R\bar{3}m$, $Z = 3$				
		T (K)	300	25
		Radiation	X-ray (Cu K_α) + Neutron (TOF)	Neutron (TOF)
		a (Å)	$7.3217(2)$	$7.333(10)$
		c (Å)	$17.298(2)$	$17.31(2)$
		B_{ov} (Å^2)	$0.2(2)$	0^*
Neutron, Bank 1	$2\theta = 9.39°$	R_{wp}	7.58	7.16
Neutron, Bank 2	$2\theta = 17.98°$		5.17	5.39
Neutron, Bank 3	$2\theta = 34.96°$		5.49	5.85
Neutron, Bank 4	$2\theta = 63.62°$		5.91	5.90
Neutron, Bank 5	$2\theta = 91.30°$		9.65	8.84
X-ray	$\lambda = 1.542$ Å		5.65	–
Dy1	$9e$, $(\frac{1}{2}, 0, 0)$	Frac. Dy	$0.94(2)$	0.94^*
		Frac. Mg	$0.06(2)$	0.06^*
Mg1	$3a$, $(0, 0, 0)$			
Mg2	$3b$, $(0, 0, \frac{1}{2})$	Frac. Dy	$0.18(6)$	0.18^*
		Frac. Mg	$0.82(6)$	0.82^*
Sb1	$9d$, $(\frac{1}{2}, 0, \frac{1}{2})$			
O1	$6c$, $(0, 0, z)$	z	$0.384(4)$	$0.383(4)$
O2	$18h$, $(x, -x, z)$	x	$0.531(3)$	$0.531(2)$
		z	$0.144(2)$	$0.144(2)$
O3	$18h$, $(x, -x, z)$	x	$0.145(3)$	$0.145(2)$
		z	$-0.055(2)$	$-0.055(2)$

Supplementary Table 1: **Structural parameters for $Dy_3Mg_2Sb_3O_{14}$.** Values of refined structural parameters for $Dy_3Mg_2Sb_3O_{14}$ determined from combined analysis of 300 K X-ray and neutron powder diffraction data, and 25 K neutron powder diffraction data. Fixed parameters are denoted by an asterisk (*).

$\mathbf{Dy_3Mg_2Sb_3O_{14}}$		
T (K)	300	25
Dy1–O1 (Å)	$2.29(3) \times 2$	$2.28(3) \times 2$
Dy1–O2 (Å)	$2.53(3) \times 2$	$2.53(3) \times 2$
Dy1–O3 (Å)	$2.46(2) \times 4$	$2.46(2) \times 4$
⟨Dy1–O⟩ (Å)	2.44	2.43
Mg1–O3 (Å)	$2.07(2) \times 6$	$2.07(2) \times 6$
Mg1–O1 (Å)	$2.01(7) \times 2$	$2.02(7) \times 2$
Mg2–O2 (Å)	$2.54(2) \times 6$	$2.538(13) \times 6$
⟨Mg2–O⟩ ()	2.41	2.41
Sb1–O2 (Å)	$1.98(2) \times 4$	$1.987(13) \times 4$
Sb1–O3 (Å)	$1.95(3) \times 2$	$1.95(3) \times 2$
⟨Sb1–O⟩ (Å)	1.97	1.97
Dy1–Dy1 (intra-plane) (Å)	$3.6609(3) \times 4$	$3.663(5) \times 4$
Dy1–Dy1 (inter-plane) (Å)	$7.1498(6) \times 6$	$7.157(6) \times 6$
Dy1–Mg2 (Å)	$3.5749(3) \times 6$	$3.579(3) \times 6$
Dy1–O1–Dy1 (°)	106.3(9)	106.7(10)
Dy1–O3–Dy1 (°)	96.4(8)	96.4(7)

Supplementary Table 2: **Bond lengths and angles in $\mathbf{Dy_3Mg_2Sb_3O_{14}}$.** Selected bond lengths and angles in $Dy_3Mg_2Sb_3O_{14}$ determined from combined analysis of 300 K X-ray and neutron powder diffraction data, and 25 K neutron powder diffraction data.

Site	Basis vector, ν	Atom, i	m_a	m_b	m_c
Dy1	1	1	2	4	4
		2	-4	-2	4
		3	2	-2	4
	2	1	-2	-4	0
		2	4	2	0
		3	-2	2	0
Mg2	1	4	0	0	12

Supplementary Table 3: **Magnetic basis vectors for $\mathbf{Dy_3Mg_2Sb_3O_{14}}$.** Basis vectors for the atoms at fractional coordinates $\mathbf{r}_1 = (\frac{1}{2}, 0, 0)$, $\mathbf{r}_2 = (0, \frac{1}{2}, 0)$, $\mathbf{r}_3 = (\frac{1}{2}, \frac{1}{2}, 0)$, and $\mathbf{r}_4 = (0, 0, \frac{1}{2})$ in the hexagonal unit cell of $Dy_3Mg_2Sb_3O_{14}$.

$Dy_3Mg_2Sb_3O_{14}$ **magnetic,** $R\bar{3}m$				
			**2-site model**	**1-site model**
		T (K)	$0.04 - 0.50$	
		Radiation	Neutron ($\lambda = 5.0\,\text{Å}$)	
		a (Å)	$7.298(3)$	$7.295(3)$
		c (Å)	$17.279(6)$	$17.277(6)$
		R_{wp}	7.54	7.63
Dy1	$9e$, $(\frac{1}{2}, 0, 0)$	C_1	$-0.30(2)$	$-0.31(2)$
		C_2	$-1.03(2)$	$-1.04(2)$
		μ_{avg} (μ_{B} per Dy)	$2.80(4)$	$2.82(4)$
Mg2	$3b$, $(0, 0, \frac{1}{2})$	C_1	$-0.08(4)$	0^*
		μ_{avg} (μ_{B} per Dy)	$1.0(5)$	0^*

Supplementary Table 4: **Magnetic-structure parameters for $Dy_3Mg_2Sb_3O_{14}$.** Values of structural parameters obtained for the two magnetic-structure models of $Dy_3Mg_2Sb_3O_{14}$ described in the text. Fixed parameters are denoted by an asterisk (*).

Site	Basis vector, ν	Atom, i	m_a	m_b	m_c
Dy1	1	1	4	0	0
		2	4	0	0
Dy2	1	3	2	0	0
		4	2	0	0
		5	2	0	0
		6	2	0	0
	2	3	0	2	0
		4	0	-2	0
		5	0	-2	0
		6	0	2	0

Supplementary Table 5: **Magnetic basis vectors for Dy_3SbO_7.** Magnetic basis vectors for the atoms at fractional coordinates $\mathbf{r}_1 = (0, 0, 0)$, $\mathbf{r}_2 = (0, 0, \frac{1}{2})$, $\mathbf{r}_3 = (x, y, \frac{3}{4})$, $\mathbf{r}_4 = (x, -y, \frac{1}{4})$, $\mathbf{r}_5 = (-x, y, \frac{3}{4})$, and $\mathbf{r}_6 = (-x, -y, \frac{1}{4})$ in the orthorhombic unit cell of Dy_3SbO_7.

Dy_3SbO_7 **magnetic,** $Cmcm$			
		T (K)	$0.6 - 4.0$
		Radiation	Neutron (TOF)
		a (Å)	$7.451358(7)$
		b (Å)	$10.533179(4)$
		c (Å)	$7.445(3)$
Bank 1	$2\theta = 9.39°$	R_{wp}	11.0
Bank 2	$2\theta = 17.98°$		9.66
Bank 3	$2\theta = 34.96°$		7.17
Bank 4	$2\theta = 63.62°$		7.09
Dy1	$4a$, $(0, 0, 0)$	C_1	$2.6(3)$
Dy2	$8g$, $(x, y, \frac{3}{4})$	x	$0.292(16)$
		y	$0.228(6)$
		C_1	$-3.3(3)$
		C_2	$2.8(3)$

Supplementary Table 6: **Magnetic-structure parameters for Dy_3SbO_7.** Refined values of magnetic-structure parameters for Dy_3SbO_7 for the magnetic-structure model described in the text. The basis vector coefficients C_ν are determined up to an overall scale factor.

Supplementary Note 1: Average magnetic structure of $Dy_3Mg_2Sb_3O_{14}$

Magnetic Bragg peaks from $Dy_3Mg_2Sb_3O_{14}$ are observed at temperatures below $0.5\,K$, but are absent at $0.5\,K$ and higher temperatures [Fig. 2b]. To isolate this magnetic Bragg scattering, we subtracted the $0.5\,K$ data from the 0.03, 0.10, 0.20, 0.30 and $0.35\,K$ data. All magnetic Bragg peaks are indexed by the propagation vector $\mathbf{k} = (0, 0, 0)$ with respect to the hexagonal unit cell of $Dy_3Mg_2Sb_3O_{14}$. Symmetry-allowed magnetic structures were determined using the program SARAh [4] and verified using ISODISTORT [5, 6]. The analysis first determines the group of symmetry elements that leave $\mathbf{k}$ invariant, and then decomposes the magnetic representations of Dy1 and Dy2 sites into irreducible representations. The decomposition of the magnetic representation for the Dy1 site is

$$\Gamma_{\mathrm{Mag}}\,(\mathrm{Dy1}) = 1\Gamma_1^{(1)} + 0\Gamma_2^{(1)} + 2\Gamma_3^{(1)} + 0\Gamma_4^{(1)} + 2\Gamma_5^{(2)} + 0\Gamma_6^{(2)}, \quad (1)$$

and the decomposition for the Mg2 site is

$$\Gamma_{\mathrm{Mag}}\,(\mathrm{Mg2}) = 0\Gamma_1^{(1)} + 0\Gamma_2^{(1)} + 1\Gamma_3^{(1)} + 0\Gamma_4^{(1)} + 1\Gamma_5^{(2)} + 0\Gamma_6^{(2)}, \quad (2)$$

where different irreps are labelled by subscript numbers (using the notation of Kovalev [7]), the dimensionality of each irrep is given by the superscript number, and the product of the superscript and the coefficient yields the number of basis vectors associated with the irrep. Rietveld refinements were performed to the $0.03 - 0.50\,K$ data to determine the average magnetic structure. In these refinements, the occupancy of the Dy site was fixed at 94%, as estimated from the crystal-structure refinements [Supplementary Figure 1 and Supplementary Tables 1 and 2]. The magnetic peak-shape parameters were modelled as Gaussian and fixed to equal the nuclear peak-shape parameters refined to 50 K data. The intensity scale factor was fixed at the value obtained from Rietveld refinement to the 50 K data, and the background was fitted by Chebychev polynomials. By testing each of the irreps Γ_1, Γ_3, and Γ_5 against the data, we found that only the Γ_3 irrep allowed a good fit. The orientation of the ordered moment of atom i is given by $\boldsymbol{\mu}_{i,\mathrm{avg}} = \sum_\nu C_\nu \mathbf{m}_{\nu,i}$; here, the C_v denotes the refined coefficient of the basis vector $\mathbf{m}_{\nu,i} = m_a^{\nu,i}\hat{\mathbf{a}} + m_b^{\nu,i}\hat{\mathbf{b}} + m_c^{\nu,i}\hat{\mathbf{c}}$, where $\hat{\mathbf{a}}, \hat{\mathbf{b}}, \hat{\mathbf{c}}$ are unit vectors parallel to the unit-cell axes. The basis vectors for this magnetic structure are shown in Supplementary Table 3. The component of the ordered magnetic moment parallel to the c-axis on the Dy1 site, $\mu_{c,\mathrm{avg}}/\mu_{\mathrm{avg}} = 0.44(3)$, shows that Dy spins are canted slightly more towards the c-axis than for cubic spin ices such as $Dy_2Ti_2O_7$, where the equivalent projection equals $1/3$.

Because Γ_3 occurs in the magnetic representation for both Dy1 and Mg2 sites, ordered moments may form on both sites in a single phase transition. The basis vectors of the Dy1 site describes an "all-in/all-out" structure, while basis vectors of the Mg2 site describe a uniform ferromagnetic component along the c-axis. Mindful of the fact that weak ferromagnetic components can be poorly determined from powder-diffraction data, we performed two separate refinements to the $0.03 - 0.50\,K$ data. First, we refine the basis-vector coefficients on both sites ("2-site refinement"); second, we constrain the magnetic moment on the Mg2 site to equal zero ("1-site refinement"). Supplementary Table 4 shows the results from each refinement. Our data are consistent with a small ordered moment ($\sim 1\mu_{\mathrm{B}}$) on the Mg2 site; however, refining this moment yields an insignificant improvement in the fit, and does not change parameter values associated with the Dy1 site. In subsequent refinements, we therefore fix $\mu_{\mathrm{avg}} \equiv 0$ on the Mg2 site for the sake of simplicity. The fits obtained to 0.10, 0.20, 0.30 and $0.35\,K$ data are shown in Supplementary Figure 8. For these refinements, we fixed the magnetic structure to the result from the refinement to $0.03 - 0.50\,K$ data, and refined only the background parameters and the overall scale factor in order to determine the temperature dependence of μ_{avg} [Fig. 3a].

Supplementary Note 2: Average magnetic structure of Dy_3SbO_7

Magnetic Bragg peaks appear in our neutron-diffraction data at $T \leq 2\,\mathrm{K}$, but are absent in $T \geq 4\,\mathrm{K}$ data. These peaks cannot be indexed by high-symmetry propagation vectors of $Dy_3Mg_2Sb_3O_{14}$, but are indexed by the propagation vector $\mathbf{k} = (0, 0, 0)$ for the Dy_3SbO_7 impurity phase [8]. We therefore identify these peaks with magnetic ordering of Dy_3SbO_7, which is reported to occur at $3.0(3)\,\mathrm{K}$ [3].

A magnetic-structure model for Dy_3SbO_7 has not been reported previously, so we turn to symmetry analysis to identify possible magnetic structures. The crystal structure of Dy_3SbO_7 (space group $Cmcm$ [8]) contains two inequivalent Dy sites, $4a$ (Dy1) and $8g$ (Dy2). The symmetry-allowed magnetic structures were determined by representational analysis using the program SARAh [4]. (irreps, Γ) of this group. The decomposition of the magnetic representation for the Dy1 site is

$$\Gamma_{\mathrm{Mag}}\,(\mathrm{Dy1}) = 1\Gamma_1^1 + 0\Gamma_2^1 + 1\Gamma_3^1 + 0\Gamma_4^1 + 2\Gamma_5^1 + 0\Gamma_6^1 + 2\Gamma_7^1 + 0\Gamma_8^1, \tag{3}$$

and the decomposition for the Dy2 site is

$$\Gamma_{\mathrm{Mag}}\,(\mathrm{Dy2}) = 1\Gamma_1^1 + 2\Gamma_2^1 + 2\Gamma_3^1 + 1\Gamma_4^1 + 2\Gamma_5^1 + 1\Gamma_6^1 + 1\Gamma_7^1 + 2\Gamma_8^1. \tag{4}$$

Because a single magnetic transition is reported in Dy_3SbO_7 [3], we consider only the irreps that appear in the decomposition for both Dy1 and Dy2 sites—namely, $\Gamma_1, \Gamma_3, \Gamma_5$, and Γ_7. We used Rietveld refinement to test each of these irreps against the $0.60 - 4.0\,\mathrm{K}$ data collected on the GEM diffractometer at ISIS [9]. In these refinements, the magnetic peak-shape parameters were modelled as convoluted pseudo-Voigt and Ikeda-Carpenter functions, and the background was fitted by a linear interpolation between between fixed points (for which the background level was subsequently refined at lower Q). The Γ_3 irrep provided the best fit-to-data, shown in Supplementary Figure 11a. This magnetic structure is shown in Supplementary Figure 11b and describes a non-collinear ferrimagnet. Because of the uncertainty associated with the wt% Dy_3SbO_7 in our sample, it was not possible to determine accurately the values of the ordered magnetic moment on Dy1 and Dy2 sites; however, the data are consistent with moment lengths on Dy1 and Dy2 sites that are equal to within $\sim$20%. The basis vectors are given in Supplementary Table 5, and refined values of magnetic-structure parameters are given in Supplementary Table 6.

Having determined a model of the magnetic structure of the Dy_3SbO_7 impurity phase, we calculated the magnetic scattering from $Dy_3Mg_2Sb_3O_{14}$ as $I = I_{\mathrm{meas}} - I_{T_{\mathrm{high}}} - I_{\mathrm{imp,Bragg}} + I_{\mathrm{imp,pm}}$, where $I_{T_{\mathrm{high}}}$ denotes a high-temperature (25 K or 50 K) measurement which is subtracted to remove non-magnetic scattering, $I_{\mathrm{imp,Bragg}}$ denotes the calculated magnetic Bragg profile for the Dy_3SbO_7 impurity, and $I_{\mathrm{imp,pm}}$ denotes the calculated paramagnetic intensity for the Dy_3SbO_7 impurity.

Supplementary References

[1] Farmer, J. M. *et al.* Structural and crystal chemical properties of rare-earth titanate pyrochlores. *J. Alloys Comp.* **605**, 63–70 (2014).

[2] Copley, J. & Cook, J. The Disk Chopper Spectrometer at NIST: a new instrument for quasielastic neutron scattering studies. *Chem. Phys.* **292**, 477 – 485 (2003).

[3] Fennell, T., Bramwell, S. T. & Green, M. A. Structural and magnetic characterization of Ho_3SbO_7 and Dy_3SbO_7. *Can. J. Phys.* **79**, 1415–1419 (2001).

[4] Wills, A. S. A new protocol for the determination of magnetic structures using simulated annealing and representational analysis (SARAh). *Physica B* **276–278**, 680–681 (2000).

[5] Stokes, H. T., Hatch, D. M. & Campbell, B. J. Isotropy software suite. URL `iso.byu.edu`.

[6] Campbell, B. J., Stokes, H. T., Tanner, D. E. & Hatch, D. M. *ISODISPLACE*: a web-based tool for exploring structural distortions. *J. Appl. Crystallogr.* **39**, 607–614 (2006).

[7] Kovalev, O. V. *Representations of the Crystallographic Space Groups* (Gordon and Breach Science Publishers, Switzerland, 1993).

[8] Siqueira, K. P. F. *et al.* Crystal structure of fluorite-related Ln_3SbO_7 (Ln = La–Dy) ceramics studied by synchrotron X-ray diffraction and Raman scattering. *J. Solid State Chem.* **203**, 326–332 (2013).

[9] Hannon, A. C. Results on disordered materials from the GEneral Materials diffractometer, GEM, at ISIS. *Nucl. Instr. Meth. Phys. Res. A* **551**, 88 – 107 (2005).
